# Invasive trophoblast promote stromal fibroblast decidualization via Profilin 1 and ALOX5

**DOI:** 10.1038/s41598-017-05947-0

**Published:** 2017-08-18

**Authors:** E. M. Menkhorst, M. L. Van Sinderen, K. Rainczuk, C. Cuman, A. Winship, E. Dimitriadis

**Affiliations:** 1 Hudson Institute of Medical Research, Clayton Vic, 3186 Australia; 2 Department of Anatomy and Developmental Biology, Monash University, Clayton Vic, 3800 Australia; 3 Department of Molecular and Translational Medicine, Monash University, Clayton, VIC 3800 Australia

## Abstract

During the establishment of pregnancy, extravillous trophoblast (EVT) must invade into the uterine decidua to facilitate decidual artery remodelling to create the placental blood supply. The local decidual environment is thought to regulate trophoblast invasion, however these interactions are poorly defined in humans. Recent evidence in women suggests impaired decidualization is associated with miscarriage and preeclampsia. Primary human endometrial stromal cells (HESC) and first trimester extravillous trophoblast (EVTs) were used to assess the effect of EVT-secreted factors on HESC decidualization, adhesion, proliferation and migration. We determined the role of profilin (PFN)1, an EVT-secreted factor, on HESC function and identified a downstream target of PFN1. EVT-secreted factors induced HESC decidualization and enhanced decidualized HESC adhesion, proliferation and migration. Recombinant PFN1 enhanced methoxyprogesterone acetate-induced HESC decidualization and proliferation. PFN1 down-regulated the expression of lipoxygenase arachidonate 5-lipoxygenase (ALOX5) in HESC and THP-1 macrophages. ALOX5 localised to decidual cells and CD68+macrophages in 1^st^ trimester decidua. This study demonstrated that EVT secretions, including PFN1, enhanced HESC decidualization and motility. This study has identified a new pathway that facilitates appropriate decidualization during the establishment of pregnancy.

## Introduction

During the establishment of pregnancy, the human blastocyst implants into the uterine endometrium to facilitate the formation of a functional placenta which is completed by the end of the first trimester. During implantation, extravillous trophoblast (EVT) invade into the uterine decidua from as early as 5 weeks gestation (~14 days after implantation)^1^. This results in the invasion and occlusion of endometrial spiral arteries by 8 weeks (begins as early as 5 weeks) and myometrial invasion by 14 weeks^1^. Such invasion is critical to create the placental blood supply which is complete at the end of the first trimester^2^. Inadequate or inappropriate implantation and placentation is thought to lead to first trimester miscarriage, placental insufficiency and other obstetric complications^3^.

The local decidual environment plays a key role in regulating trophoblast invasion^2, 4^. Prior to implantation and in preparation for pregnancy, stromal cells of the uterine endometrium become ‘decidualized’. Decidualization describes the proliferation and differentiation of endometrial stromal cells (ESC) into decidual cells. It is a slow process which involves the categorical reprogramming of ESC such that different genes are expressed at different stages of the differentiation process^5^. In women, decidualization occurs spontaneously in stromal cells adjacent to spiral arterioles during the mid-secretory phase of the menstrual cycle under the physiological stimulus of progesterone, regardless of the presence of a blastocyst^6^. If implantation occurs, decidualization intensifies and continues to form the decidua of pregnancy. This is in contrast to rodents where decidualization begins post-implantation.

Recent evidence in women demonstrates that decidualization is impaired prior to the onset of symptoms in recurrent miscarriage, preeclampsia (PE) and placenta accreta suggesting abnormalities in decidualization contribute to the development of these disorders^7–9^. While it is known that locally produced decidual factors act in association with progesterone to drive decidualization^10^ there is very little known of decidual-EVT interactions in humans.

The effect of EVTs on HESC function has only limited research to date. HESC treated with EVT cell surface and secreted factors^11, 12^, show altered HESC cytokine and angiogenic factor mRNA expression, including IL8, and the decidual marker IGFBP1. When co-cultured with 1^st^ trimester placental explants, term decidual fibroblasts maintained their decidualization compared to control (no treatment)^13^, however this likely reflects progesterone secreted by the placenta itself. While EVTs secrete progesterone^14^, it is not known whether EVTs themselves would affect HESC decidualization. Finally, co-cultured non-decidualized and decidualized HESC with the AC1M88 trophoblast cell line enhances HESC migration^15^, however this has not been confirmed using primary first trimester trophoblast.

We previously identified profilin 1 (PFN1) as secreted by EVTs^16^. PFN1 is an actin-binding protein which is also found extracellularly, including in serum^17^ and conditioned media (CM)^16, 18^. The function of extracellular PFN1 is not known. *In vitro*, EVT secretion of PFN1 is enhanced following treatment with decidualized HESC CM^16^, however the role of EVT-secreted PFN1 is not understood.

We hypothesized that EVT factors regulate HESC decidualization in first trimester decidua. We investigated the effect of EVT-secreted factors and PFN1 on HESC function (decidualization, adhesion, proliferation and migration). PFN1 targets in HESC and THP-1 macrophage cells were also investigated.

## Results

### EVT CM induced HESC decidualization

**Figure 1:**
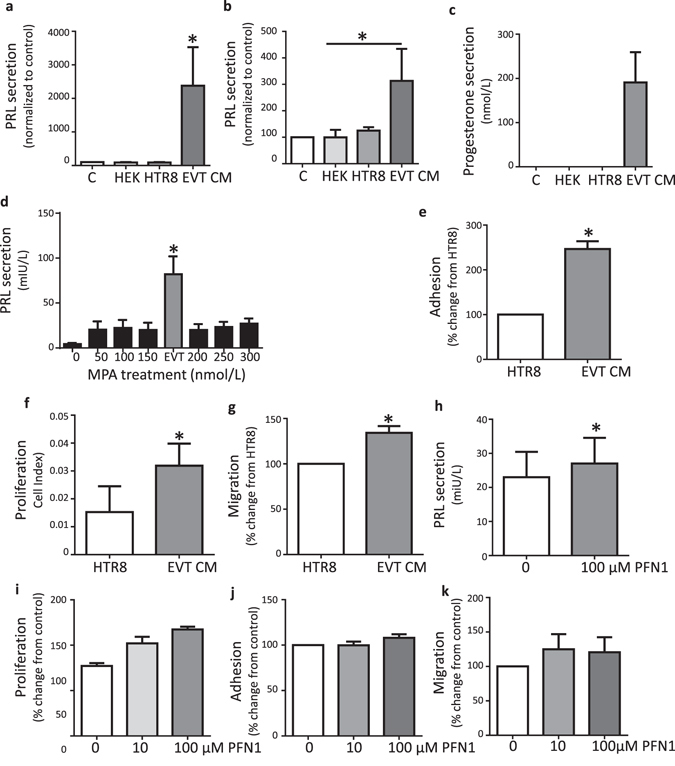
EVT CM enhanced HESC decidualization via profilin 1 (PFN1). (**a**) HESCs (n = 4) were treated with E for 14 days and CM treatments (Hek293, HTR8[/SVneo], EVT) included from day 7. PRL secretion was measured on day 14. PRL secretion was significantly elevated by factors in EVT CM compared to media alone or cell line control CMs. (**b**) HESCs (n = 4) were treated with E+MPA for 14 days with CM treatments (Hek293, HTR8, EVT) included from day 7. PRL secretion was measured on day 14. PRL secretion was elevated in HESC treated with EVT CM compared to Hek293 control CM but not significantly changed compared to media alone or HTR8/SVneo CM. (**c**) Only EVT CM contained progesterone (n = 5). (**d**) PRL secretion by HESC was significantly higher when treated with EVT CM compared to a range (0–300 nmol/L) of MPA concentrations (n = 3/group). (**e–k**) HESCs were decidualized with E+MPA for 12 days before being subjected to functional assays with CM or PFN1 treatments. (**e**–**g**) Decidualized HESC adhesion (e, n = 3), proliferation (f, n = 4) and migration (g, n = 6) were significantly enhanced by treatment with EVT CM. (**h**) PFN1 treatment during decidualization (days 7–14 of E+MPA treatment) significantly increased HESC PRL secretion on day 14 (h, n = 4). **i**. PFN1 treatment significantly enhanced proliferation of decidualized HESC (n = 3). (**j,k)** PFN1 treatment had no effect on decidualized HESC adhesion (j; n = 3) or migration (k; n = 4). Data are mean ± SEM, *p < 0.05, significant difference from control; ^p < 0.05 significant difference between treatments labelled with ^. a,b,d: Friedman test; d,e,f,g,h: paired t-test; i, j,k: repeated measures one-way ANOVA.

To determine whether EVT CM induced HESC decidualization we treated HESC with oestrogen (E) (non-decidualized HESC) or E+ methoxyprogesterone acetate (MPA) (decidualizing HESC) for 6 days, then EVT or control cell line (Hek293 [kidney epithelial cell line] or HTR8SV/neo [1^st^ trimester EVT-derived cell line]) treatments at 50% final volume were added to the hormone treatments for a further 8 days. As the induction of PRL secretion was highly variable between women (Supplementary Figure), this data (Fig. 1a,b) is shown as normalized to E or E+MPA alone controls.

EVT CM alone induced HESC decidualization (HESC treated with E alone; measured by PRL secretion; Fig. 1a). In decidualizing HESC (HESC treated with E+MPA), EVT CM significantly enhanced decidualization compared to HEK293 CM but had no significant effect compared to control or HTR8SV/neo CM (Fig. 1b). Since PRL was used as a measure of decidualization, PRL was assayed in EVT and cell line CM used to treat HESCs. PRL was undetectable in EVT, HTR8/SVneo and HEK293 CM (n = 4 pooled samples).

Few factors are known to be capable of inducing decidualization beyond progesterone and cyclic adenosine monophosphate. EVTs are known to secrete progesterone^14^ and here EVT CM contained progesterone (Fig. 1c) at levels 3.8-fold higher (190.9 ± 68.6 nmol/L) than the concentration (50 nmol/L) of MPA added to cells to stimulate decidualization, suggesting EVT-secreted progesterone induced decidualization of HESCs. However, when HESC were induced to decidualize using E+MPA for 14 days at a variety of MPA concentrations (50–300 nmol/L), there was no change in PRL secretion across the MPA concentration range (Fig. 1d). Moreover, HESC treated with MPA at all concentrations showed significantly lower PRL secretion compared to HESC treated with EVT CM (Fig. 1d), suggesting EVT CM contained factors other than progesterone which synergized to enhance decidualization.

### EVT CM enhanced decidualized HESC adhesion, proliferation and migration

All functional experiments were performed using HESC decidualized for 12 days by E+MPA treatment to mimic the decidualization that occurs *in vivo* prior to blastocyst implantation and subsequent EVT invasion. Decidualization treatments were included in the media during functional assays. As control and HEK293 CM had no effect on decidualization (Fig. 1a,b) HTR8/SVneo CM was chosen as a control CM for all subsequent experiments. HTR8 CM was chosen as a control over media alone as factors in the media (including fetal calf serum) would have been metabolised in CM but not media alone.

#### EVT CM enhanced decidualized HESC adhesion, proliferation and migration

Decidualization involves the proliferation and differentiation of HESC. EVT CM significantly enhanced decidualized HESC adhesion (Fig. 1e) and proliferation after 48 h (Fig. 1f).

As previously published^15^, we also found that only decidualized HESC (not non-decidualized HESC) are motile. Therefore we investigated whether EVT secreted factors regulated decidualized HESC motility. EVT CM significantly enhanced decidualized HESC migration (Fig. 1g) compared to HTR8/SVneo CM control.

### EVT-secreted PFN1 enhanced HESC decidualization

We previously localized PFN1 to EVTs, glandular epithelium and leukocytes in 1^st^ trimetester decidua basalis^16^. We demonstrated that EVTs secrete PFN1 *in vitro* and that this is elevated in response to treatment with decidualized HESC CM^16^. Importantly, PFN1 does not localize to decidual cells in 1^st^ trimester implantation sites^16^ and HESC induced to decidualize *in vitro* did not secret detectable PFN1 (non-decidualized or decidualized; data not shown). Therefore, we investigated whether EVT-secreted PFN1 could act on HESC to promote decidualization.

#### PFN1 promoted HESC decidualization

HESC were induced to decidualized for 14 days by treatment with E+MPA with PFN1 or control treatment included at every media change. PFN1 treatment significantly enhanced decidualization in HESC treated with E+MPA (Fig. 1h) but had no effect on decidualization in HESC treated with E alone (data not shown).

#### PFN1 promoted decidualized HESC proliferation

HESC were decidualized by E+MPA treatment for 12 days before PFN1 treatment was included in experimental conditions. PFN1 treatment did not significantly increase proliferation in decidualized HESC at 72 h (Fig. 1i) and similarly had no effect on decidualized HESC adhesion or migration (Fig. 1j,k).

### PFN1 acts via ALOX5 to regulate decidualization

#### PFN1 decreased ALOX5 mRNA expression in HESC

To determine the mechanism by which PFN1 enhanced HESC decidualization, we utilized a Human Hypertension array to identify factors in HESC regulated by PFN1. HESC were decidualized for 10–12 days with E+MPA before a 6 h treatment with PFN1 or control and RNA collection for the array. Samples (n = 4) were pooled for the array.

**Figure 2:**
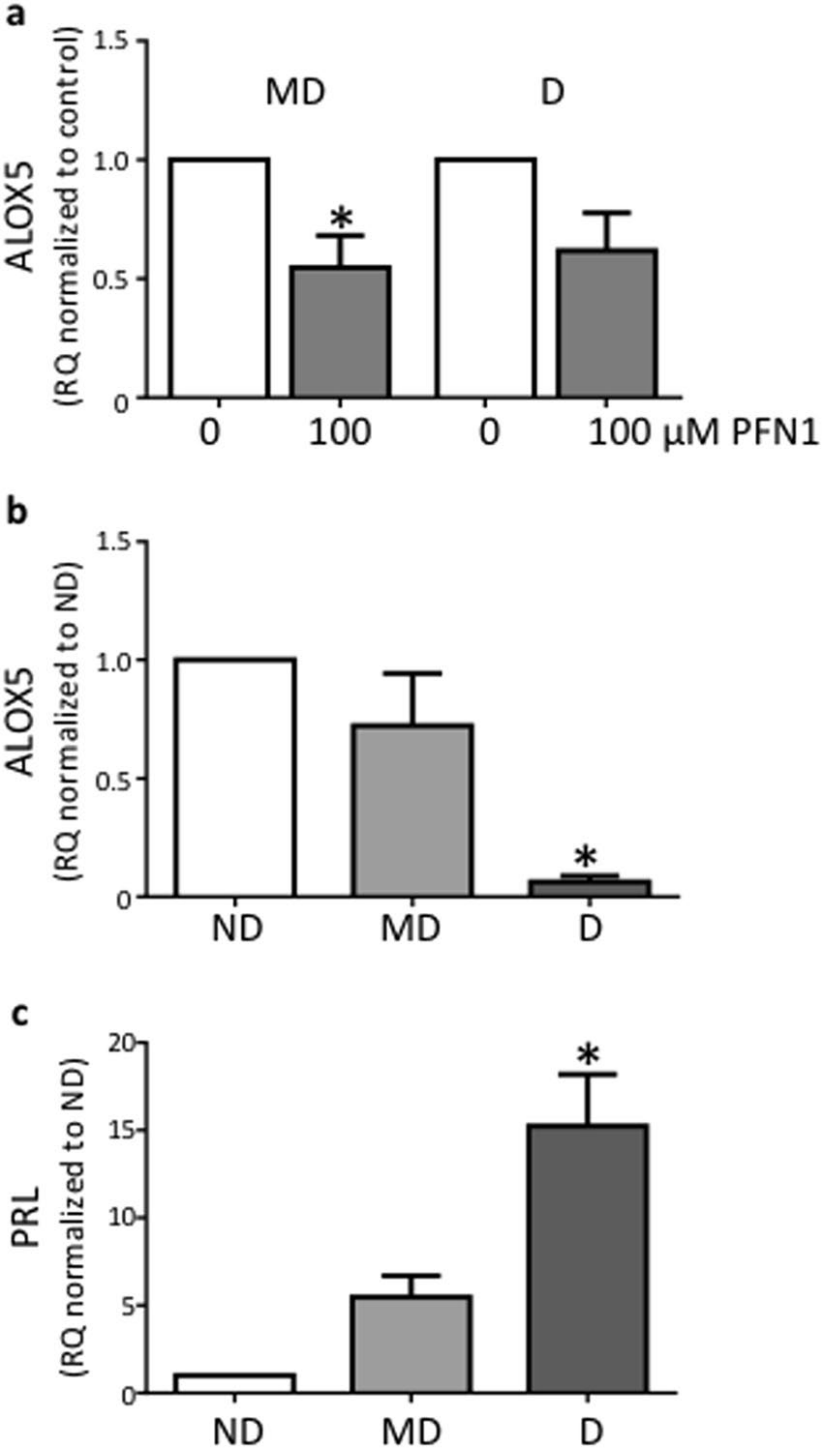
Exogenous profilin 1 decreased HESC ALOX5 expression. (**a**) PFN1 significantly decreased ALOX5 mRNA in minimally decidualized (MD) HESC (decidualized for 2 days, do not secrete detectable PRL; n = 4–6/group) but had no significant effect on decidualized (D) HESC (decidualized for 10–12 days, secrete PRL). (**b**) ALOX5 mRNA expression declined during *in vitro* HESC decidualization (n = 4–10/group). (**c**) Prolactin confirmation of decidualization (n = 4–10/group). mRNA expression was determined by semi-quantitative PCR normalized to 18S. Data are mean ± SEM, *p < 0.05, significant difference from control. a: paired t-test; b,c: one-way ANOVA.

ALOX5, a lipoxygenase involved in the derivation of bioactive lipoxins from arachidonic acid^19^, was identified as a potential target of PFN1 by this array (Table 1 and Supplementary Table). The effect of PFN1 on ALOX5 production was investigated in both minimally decidualized HESC (decidualized with E+MPA for 2 days but which did not yet secrete detectable levels of prolactin), and decidualized HESC (decidualized with E+MPA for 12 days and secreted prolactin). Real-time PCR confirmed ALOX5 mRNA was significantly downregulated by PFN1 in minimally decidualized HESC but not in decidualized HESC (Fig. 2a). Supporting a role for ALOX5 in decidualization, ALOX5 was down-regulated during *in vitro* decidualization (Fig. 2b). Decidualization was confirmed by PRL mRNA expression (Fig. 2c).

**Table 1:** Genes altered by PFN1 (>2-fold) in decidualized (treated with E+MPA for 10–12 days) HESC stimulated with 100 µM PFN1 for 6 h.

		PFN1 treated vs. Control	
		Fold Change	p-value
Upregulated genes			
MYLK2	Myosin light chain kinase 2	2.5757	0.242757
Downregulated genes			
ADRB1	Adrenergic, beta-1-, receptor	−3.7308	0.242764
ALOX5	Arachidonate 5-lipoxygenase	−4.2358	0.083988
CNGA2	Cyclic nucleotide gated channel alpha 2	−2.2372	0.257212
DRD5	Dopamine receptor D5	−3.519	0.56862
NOSTRIN	Nitric oxide synthase trafficker	−2.6417	0.346766
NPPC	Natriuretic peptide C	−2.286	0.215343
UTS2R	Urotensin 2	−3.0634	0.12733

#### ALOX5 immunolocalised to first trimester decidual and immune cells

**Figure 3:**
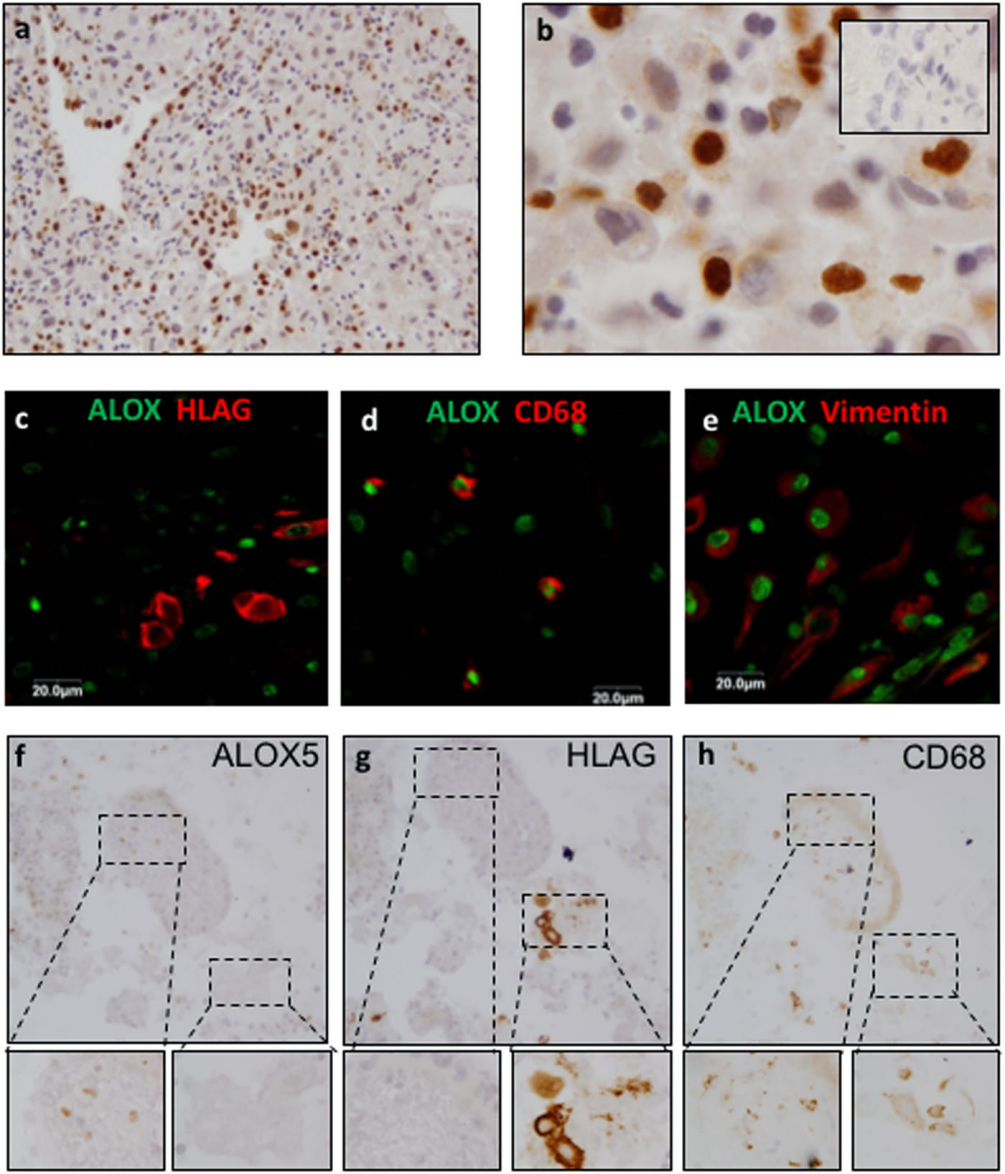
ALOX5 inmmunolocalization in first trimester decidua. (**a–b)** ALOX5 showed nuclear staining in first trimester decidua basalis. (**c–e)** Immunoflorescence showed ALOX5 did not co-localize with (**c**). HLAG (EVT marker) but did co-localize with (**d**). CD68 (macrophage marker) and (**e**). Vimentin (HESC marker). ALOX5 - green, CD68, Vimentin and HLAG – red. (**f–h**) Serial sections of first trimester decidua basalis showing that ALOX5 (**f**) was not present in tissue surrounding EVTs (determined by presence of HLAG; (**g**). The presence of macrophages in this tissue was confirmed by CD68 staining (**h**). Insert shows negative control staining.

We have previously localized PFN1 to EVTs and the glandular epithelium within the decidua basalis^16^. ALOX5 localized to cells within the decidua (Fig. 3a), showing predominantly nuclear localization (Fig. 3b). Immunofluorescence on first trimester placenta showed ALOX5 did not co-localise with HLAG (an EVT marker) (Fig. 3c) however co-localization identified ALOX5 production in macrophages (CD68 positive) (Fig. 3d) and stromal cells (vimentin positive) (Fig. 3e). Analysis of ALOX5 using serial sections showed that ALOX5 was absent in sections of decidua where EVTs were located (HLAG staining) (Fig. 3f,g) but was unaffected by the presence of macrophages (CD68 staining, Fig. 3h), supporting our *in vitro* observation that EVT-derived PFN1 may reduce ALOX5 production.

#### PFN1 and ALOX5 production in human endometrium across the menstrual cycle

**Figure 4:**
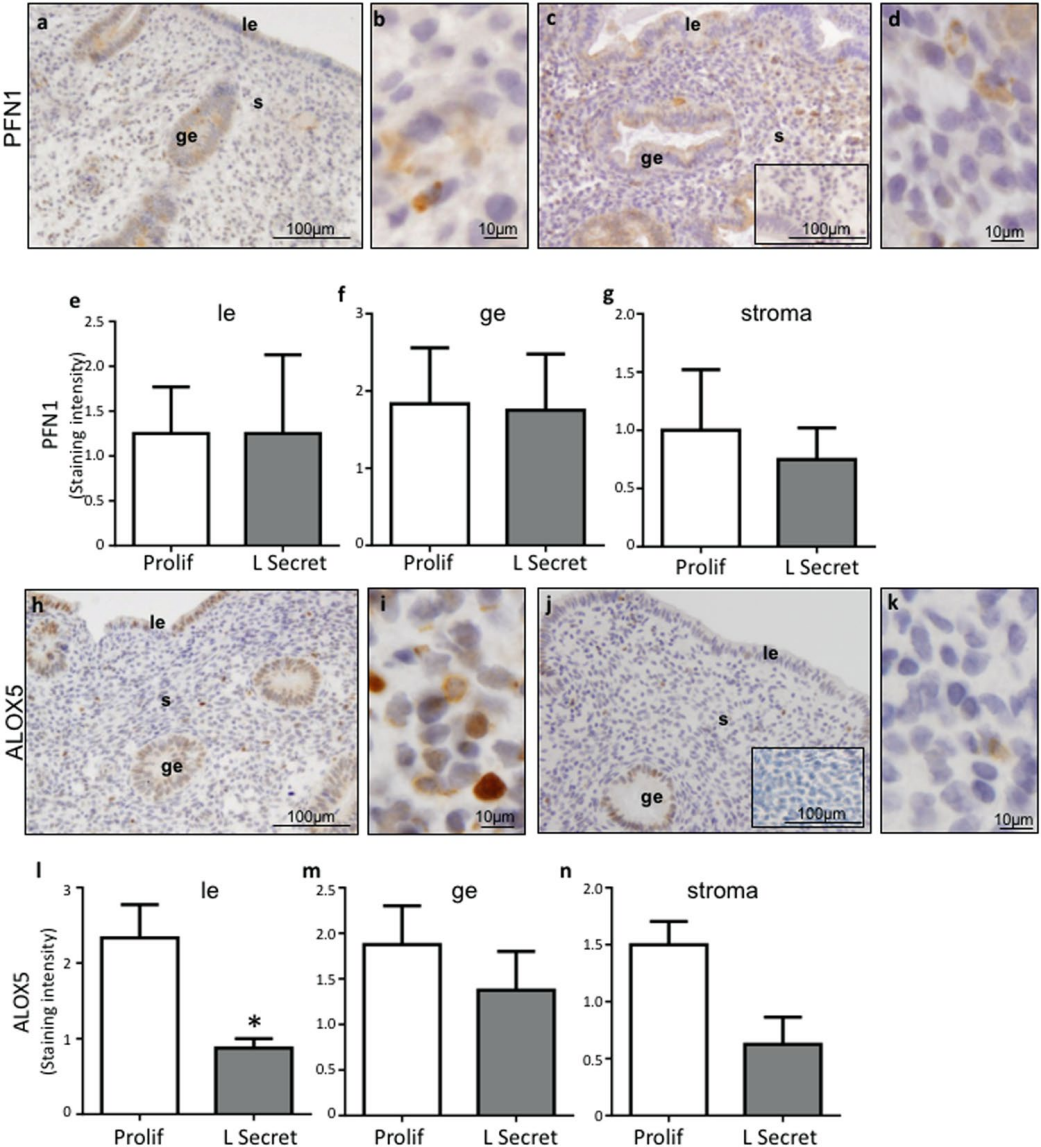
PFN1 and ALOX5 immunolocalization in endometrium. (**a–d)** Representative photomicrographs of endometrial biopsies showing localization of PFN1 protein in the stroma (s), luminal (le) and glandular (ge) epithelium in the proliferative (**a,b**) and late secretory (**c,d**) phases. (**e–g)** PFN1 immunostaining intensity scoring in the luminal epithelium (e, n = 3), glandular epithelium (f, n = 3) and stroma (g, n = 3). (**h–k**) Representative photomicrographs of endometrial biopsies showing localization of ALOX5 protein in the stroma, luminal and glandular epithelium in the proliferative (**h–i**) and late secretory (**j–k**) phases. (**l–n**) ALOX5 immunostaining intensity scoring in the luminal epithelium (l, n = 4), glandular epithelium (m, n = 4) and stroma (n, n = 4). Inserts show negative staining. Data are mean ± SEM, *p < 0.05, significant difference from proliferative phase; e-g;l-n: Mann Whitney t-test.

PFN1 immunolocalized to the luminal and glandular epithelium c and showed slight staining in the endometrial stroma (Fig. 4a–d). PFN1 staining was not changed throughout the menstrual cycle in any cellular compartment of the endometrium (Fig. 4e–g; data for early and mid-secretory stages not shown). ALOX5 protein immunolocalized to the luminal and glandular epithelium and stromal compartments of the endometrium (Fig. 4h–k) in both the proliferative (Fig. 4h,i) and late secretory (Fig. 4j,k) stages of the menstrual cycle. ALOX5 immunostaining was significantly reduced in the luminal, but not the glandular epithelium or the stroma, in the late secretory phase compared to the proliferative phase of the menstrual cycle (p < 0.05, Fig. 4l–n). Overall, given the very minimal PFN1 staining in the stroma, and the fact that PFN1 and ALOX5 staining did not correlate in the cycling endometrium, this data suggests that endometrial expressed PFN1 likely does not regulate decidualization or ALOX5 production during the menstrual cycle.

#### ALOX5 is decreased by PFN1 in human monocyte cell line (THP-1)

**Figure 5:**
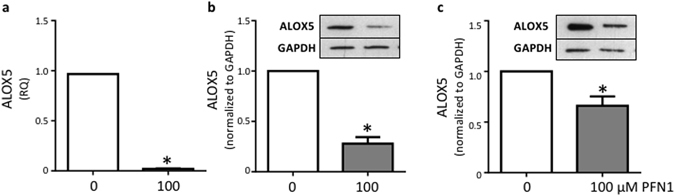
Exogenous profilin 1 decreased THP-1 ALOX5 expression. (**a**) ALOX5 mRNA was decreased after 24 hr treatment with PFN1 (100 μM) in THP-1 cells (n = 4). ALOX5 protein was decreased after (**b**) 48 h (n = 3) and (**c**) 72 hr (n = 3) of PFN1 (100 µM) treatment on THP-1 cells determined by Western blot normalized to GAPDH. mRNA expression was determined by semi-quantitative PCR normalized to 18S. Data are mean ± SEM, *p < 0.05, significant difference from control. a-c: student’s t-test.

ALOX5 is expressed primarily in leukocytes^19^, therefore we investigated if PFN1 altered ALOX5 levels in the human monocyte cell line, THP-1. PFN1 treatment significantly reduced ALOX5 mRNA expression in THP-1 cells after 24 hr (Fig. 5a) and protein levels after 48 and 72 hr (Fig. 5b,c).

## Discussion

**Figure 6:**
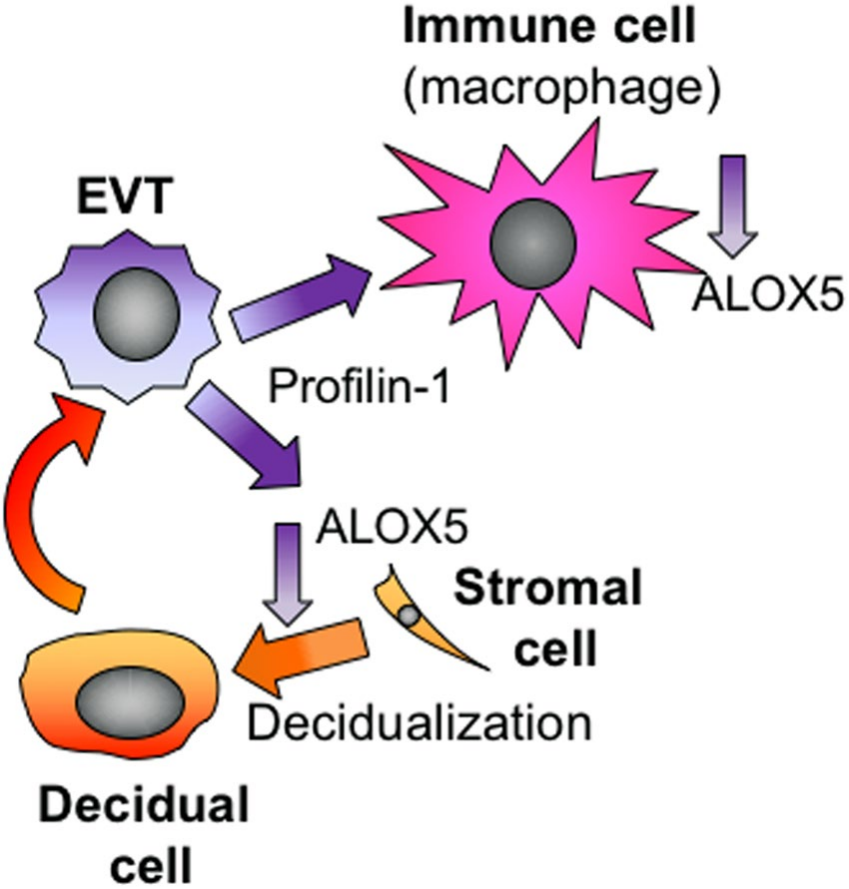
EVT-decidual-immune cell interaction. EVT-HESC synchrony and cross-talk is critical for implantation during early pregnancy. Decidualized HESC factors promote EVT secretion of profilin 1, which acts to promote stromal cell decidualization via the down-regulation of ALOX5. EVT-secreted profilin 1 also down-regulates ALOX5 in macrophages where it likely regulates cytokine production.

This study identifies the importance of HESC-EVT crosstalk in facilitating decidualization during the establishment of pregnancy (Fig. 6). It identified for the first time that EVT-secreted factors induced HESC decidualization and identified PFN1 as a novel factor that enhanced progesterone-induced decidualization *in vitro*.

For the first time we have investigated the effect of EVT soluble factors on HESC differentiation. Given the importance of decidualization to EVT invasion^20^, it is perhaps unsurprising that EVT released factors enhanced HESC decidualization. EVT-secreted progesterone^14^ likely induced HESC decidualization, however our data demonstrated that MPA at an equivalent concentration to EVT-secreted progesterone was insufficient to drive decidualization to the degree seen following treatment with EVT CM, and that additional EVT factors, including PFN1 contributed to progressing decidualization. However, by comparison to EVT sections, PFN1 alone had a modest effect on prolactin secretion and therefore decidualization. It is well known that there are a wealth of factors that regulate decidualization of human endometrial stromal cells. Moreover, decidualization is regulated in a tightly co-ordinated manner and an imbalance of the co-ordinated process may result in abnormal decidualization leading to placental insufficiency and associated pregnancy disorders. Our data is in agreement with previous studies where numerous factors were identified in EVT CM known to be associated with decidualization^16^ including Plasminogen activator inhibitor 1 and Thioredoxin.

PFN1 is an actin-binding protein which is also found extracellularly, including in serum^17^ and CM^16, 18^. Our data supports an extracellular role for PFN1 in EVT-decidual-macrophage cross-talk. The mechanism behind PFN1 release is not well understood; it does not have a signal motif, however it has been detected in dendritic-derived exosomes^18^. While there is no known cell surface receptor for PFN1, exogenous PFN1 activates ERK1/2, Akt, p70 as well as increasing AP-1 DNA binding activity and c-jun expression^17, 21^. Human toll like receptor (TLR) 5 acts as a receptor to a profilin-like molecule produced by *Toxoplasmosis gondii* ^22^, but this has not been confirmed using mammalian profilin. However, *T. gondii* profilin stimulates IL8 in HEK293 cells via TLR5^22^: HESC production of IL8 is upregulated by co-culture with trophoblast cells^12^ or treatment with trophoblast CM^11^. It remains to be determined whether mammalian PFN1 binds to TLR5 and whether PFN1 stimulates IL8.

Little is known of the function of extracellular PFN1. Exogenous recombinant PFN1 stimulates proliferation and migration of human coronary vascular smooth muscle cells^17^. For the first time, we showed that PFN1 enhanced decidualization and proliferation of HESC, likely via down-regulating ALOX5. As ALOX5 was down-regulated during MPA-induced *in vitro* decidualization and PFN1 is absent in cycling endometrium, we suggest that EVT-secreted PFN1 is an additional factor expressed by EVTs to downregulate ALOX5 during the early phase of the decidualization process (Fig. 6).

ALOX5 is a lipoxygenase enzyme which converts arachidonic acid to leukotrienes^19^. Leukotrienes have roles in normal host defence and disease states with acute or chronic inflammation as part of the normal pathophysiology^19^. ALOX5 is recognised for its role in the formation of proinflammatory leukotrienes, although more recently it has been recognized for its role in the biosynthesis of products which have anti-inflammatory functions^19^. Imbalance between pro- and anti-inflammatory cytokines can lead to aberrant inflammation which is often seen in pregnancies with complications including preeclampsia^23^. The precise role of ALOX5 in the decidua is currently unknown. In a number of studies, inhibition of ALOX5 down-regulates the expression of pro-inflammatory cytokines including the preeclampsia associated cytokines TNFα^24^, IL6^24^ and IL1β^25^, and up-regulates the expression of anti-inflammatory IL10^25^. These pro-inflammatory factors are known to activate macrophages, leading to the cascade of macrophage-induced EVT apoptosis and impaired invasion proposed in preeclampsia^26^.

PFN1 expression is altered in inflammatory conditions including atherosclerosis^17, 27^. This study provides the first evidence that extracellular PFN1 may have a role in inflammation via its regulation of ALOX5 and that. EVT-secreted PFN1 suppresses decidual and macrophage ALOX5 during the establishment of pregnancy. Further work is required to determine whether PFN1 suppression of ALOX5 in decidua or macrophages would likewise inhibit pro-inflammatory cytokine production during early pregnancy.

## Conclusion

This study highlights the importance of EVT-HESC cross-talk and synchrony to promote normal decidualization facilitating EVT invasion and the successful formation of a functional placenta. We provide evidence of a novel signalling pathway that promotes decidualization during early pregnancy.

## Methods

### Ethics approval and consent to participate

This study was approved by the Monash Health Human Research and Ethics Committee (#09317B; #06014C). Written and informed consent was obtained from each patient before surgical intervention. Methods were carried out in accordance with Monash Health Human Research Guidelines.

### Primary tissue collection and isolation and cell culture

#### EVT isolation

Normal first trimester placental tissue (n = 15) was collected from healthy women undergoing termination of pregnancy for psychosocial reasons (amenorrhea 6–12 weeks). EVT were isolated from normal first trimester placental tissue as previously described^16^. The purity of primary cytotrophoblasts was confirmed by immunocytochemistry for HLAG (BD Biosciences, CA, USA) as previously described^16^.

#### HESC isolation

Endometrial biopsies (n = 36) were collected by dilatation and curettage from fertile women (aged 18–40 years) scheduled for tubal ligation or undergoing testing for tubal patency during days 8–21 of a normalised 28 day menstrual cycle. Tissues were assessed by a pathologist and had no obvious endometrial pathology. The women had no steroid treatment or other medication for at least 3 months before the collection of tissue.

HESC were isolated by collagenase digestion and filtration as described previously^28^ except that the collagenase used was from Sigma (Type III C0255). This method results in a 97% pure stromal cell culture^28^.

#### Culture conditions

**EVTs** were maintained in DMEM/F12 (Gibco) containing 10% FCS (Gibco) and 1% antibiotics (penicillin, streptomycin, amphoceterin B; Gibco). EVTs were cultured on Growth-factor Reduced Matrigel (Corning) diluted 1:5 in serum-free DMEM/F12 to promote differentiation towards an EVT phenotype.

**HESC** were cultured on plastic in DMEM/F12 containing 10% charcoal-stripped FCS (Gibco) and 1% antibiotics prior to experimental use.

**HEK293** are an embryonic kidney epithelial cell line (ATCC). HEK293 cells were maintained in EMEM (Invitrogen) containing 10% FCS and 1% antibiotics.

**HTR8/SVneo** (a gift from Charles Graham; authenticated 16/06/2016; MHTP Medical Genomics Facility; RRID:CVCL_7162) are a 1^st^ trimester EVT derived cell line. HTR8/SVneo cells were maintained in RPMI (Invitrogen) containing 10% FCS and 1% antibiotics.

**THP-1** cells (human monocyte cell line non-adherent; authenticated 16/06/2016; MHTP Medical Genomics Facility; RRID:CVCL_0006) were cultured in RPM1 containing 10% FCS and 1% antibiotics, before stimulated towards an adherent, macrophage phenotype by overnight treatment with phorbol 12-myristate 13-acetate (PMA; 12.5 ng/ml).

All cells were cultured at 37 °C, 20% O_2_ in a 5% CO_2_ humidified culture incubator.

CM collection Isolated primary first trimester EVT, HTR8/SVneo and HEK293 cells were cultured on 1:5 diluted growth-factor reduced Matrigel in 10% FCS DMEM/F12. CM was collected after 48 h. EVT CM was pooled (n = 5/group) from EVTs. HTR8/SVneo and HEK293 CM was pooled from n ≥ 3.

### HESC decidualization

HESC (cultured from individual endometrial biopsies) were grown to confluence (maximum time to confluence 5 days) and decidualized by treatment with oestradiol (E, 10^−8^ M, Sigma) and medroxyprogesterone acetate (MPA, 10^−7^ M, Sigma) for 12–14 days as previously described^16^, except no cyclic adenosine monophosphate was included. Media (including treatments below) was replaced every 2–3 days. The level of PRL secretion taken as a measure of decidualization^28, 29^.

HESC were defined as non-decidualized (treated with E alone, do not secrete PRL); minimally decidualized (treated with E plus MPA for 2 days, do not secrete detectable PRL); or decidualized (treated with E plus MPA for 12–14 days, secrete PRL).

#### EVT CM and profilin-1 (PFN1) induced HESC decidualization

- EVT, HEK293 or HTR8/SVneo CM (50% final volume) was included from day 7-day 14 of decidualization.
- Recombinant human PFN1 (100 µM; Abcam #ab87760) or vehicle control was included from day 0 - day 14 of decidualization. The PFN1 concentration was determined by pilot studies (range 0.1–100 µM).

### PRL assay

Prolactin (PRL) secretion by HESC was assayed by ELISA (as previously described 16: Bioclone Aust. Pty Ltd, NSW, Australia or conducted by Monash Health Pathology Australia, Monash Medical Centre).

### Progesterone assay

Progesterone was assayed by competitive binding immunoenzymatic assay using a rabbit anti-human progesterone antibody performed on a Beckman Coulter Unicel DXI 800 analyzer conducted by Monash Pathology (Monash Medical Centre, Clayton, Victoria, Australia). Analytical range: 0.3–127.2 nmol/L. Coefficient of variation 6.4%.

### Functional assays

#### xCELLigence experiments

The effect of EVT CM (FV 50%) and/or PFN1 (0.1–100 µM) on decidualized HESC (HESC pre-treated with E+MPA for 12 days) adhesion, proliferation and migration was assessed using the xCELLigence real-time cell analyser (John Morris) as previously published ^30^.

#### xCELLigence adhesion and proliferation

HESC decidualized using E+MPA as described above seeded on day 12 at 10,000 cells/well in 2% csFCS DMEM/F12 including E+MPA. Adhesion and proliferation were assessed using the E plate as previously described^30^. The plate was monitored once every 15 secs for the first 6 h to monitor adhesion then every 15 minutes until 72 h to monitor proliferation. Data was calculated with RTCA software 1.2.1 and exported for statistical analysis. EVT or HTR8/SVneo CM was included at 50% FV; PFN1 treatments were included at 10 and 100 µM.

#### xCELLigence migration

HESC decidualized using E+MPA as described above before being seeded on day 12 at 30,000 cells/well in 2% csFCS DMEM/F12 including E+MPA. Migration was assessed using the CIM plate as previously described^30^; The plate was monitored every 15 min for 17 h (migration). EVT or HTR8/SVNeo CM was included at 50% FV; PFN1 treatments were included at 10 and 100 µM. Treatments were included in the upper well; the bottom container contained 10% FCS. Data was calculated with RTCA software 1.2.1 and exported for statistical analysis

### Immunohistochemistry

Immunohistochemistry was performed on formalin-fixed first trimester decidua basalis sections (4μm) as previously described^31^. Slides were dewaxed, rehydrated and antigen retrieved in 0.01 M sodium citrate before quenching endogenous peroxidase activity. Non-specific binding was blocked with 10% normal goat serum and 2% normal human serum before the primary antibody was applied overnight at 4 °C. Antibody localization was detected by sequential application of biotinylated goat anti-rabbit IgG or horse anti-mouse IgG (1:200) followed by Vectastain ABC Elite kit (Vector). Staining was visualized by the diaminobenzidine substrate (DakoCytomation), tissues were counterstained with Harris hematoxylin (Sigma–Aldrich) and mounted. Representative images were taken using CellSense software.

Immunostaining intensity in the endometrium was analysed semiquantitively by two independent and blinded observers as previously described^32^. The intensity of PFN1 staining in the endometrium was assessed and allocated a score between 0 (no staining) and 3 (strong staining) relative to positive and negative controls.

### Immunofluorsecence

Formalin-fixed sections were treated as described for immunohistochemistry with modifications as follows: non-immune serum and 10% CAS block diluted in PBS/0.1% bSA and washes performed in 0.6% Tween20 in PBS/0.1% bSA; secondary antibody incubation (Donkey α-rabbit alexa fluor 488, Donkey α-mouse alexa fluor 568 and Donkey α-goat alexa fluor 568; at 1:400) in non-immune serum for 2 h and then sections were mounted using Vectastain (DAKO).

#### Antibodies used

ALOX5 (Abcam #ab169755; IHC and IF: 1:100), CD68 (DAKO M0814 [RRID:AB_2314148]; IHC: 1:200; IF: 1:100), human leukocyte antigen G (HLAG) (BD Pharmingen cat#557577 [RRID:AB_396753]; IHC: 1:500; IF: 1:200), PFN1 (Santa Cruz Biotech #sc-137236 [RRID:AB_2163203]; IHC 1:400), vimentin (Santa Cruz Biotech #sc-6260 [RRID:AB_628437]; IF: 1:100). Negative control isotype rabbit, goat or mouse IgG (Dako) was included for every tissue section.

### PFN1 treatment for RNA and protein extraction

Adherent THP-1 cells and HESC (non-decidualized [no treatment], minimally decidualized [treated with E+MPA for 2 days] or decidualized [treated with E+MPA for 10–12 days]) were treated with PFN1 (100 µM) or vehicle control (PBS) for 6hrs (array), 24hrs (RNA) or 48 and 72hr (protein).

#### RNA preparation and quantitative Real time RT-PCR

Total RNA was isolated and reverse transcribed (250 ng) as previously described^31^ using TRI Reagent RNA Isolation system (Sigma #T9424) following manufacturer’s protocol. All samples were DNase I treated (DNAfree^TM^, Ambion, Foster City, CA, USA) and concentrations were quantified using the NanoDrop1000 (Thermo, Willmington, USA). Human Hypertension RT2 Profiler PCR Arrays (QIAGEN #PAHS-037Z) were performed in accordance with manufacturer instructions on decidualized HESC only. For qPCR total RNA was reverse transcribed using Superscript III First-Strand Synthesis System (Invitrogen) according to the manufacturer’s protocol. Real time RT–PCR analyses were performed on the ABI 7500HT fast block real time PCR system (Applied Biosystems, Foster City, CA, USA) in triplicate in 384-well Micro Optical plates (Applied Biosystems) with the Power SYBR green master mix (Applied Biosystems) and 200 nM primers.

The PCR protocol was as follows: 95 °C for 10 min and 40 cycles of 95 °C for 15 s followed by 60 °C for 1 min. Relative expression levels were calculated by the comparative cycle threshold method (ΔΔCt) as outlined in the manufacturer’s user manual, with 18S ribosomal RNA serving as the endogenous control for normalization. Products were sequenced to confirm specificity.

The sequences for the ALOX5, Prolactin, and 18S primers (200 nM used in reaction) were as follows;

*ALOX5* Fwd-GTTCCAGTGACTTCCACGTCCA, Rev-GAGGCCACACTCGCAGATGA; *Prolactin* Fwd-GGTTCATCCTGAAACCAAAG, Rev-CTTCAGGAGCTTGAGATAATTG; *18s* Fwd- GATCCATTGGAGGGCAAGTCT, Rev-CCAAGATCCAACTACGAGCTT.

#### Western Blot

THP-1 cells were collected after 48 or 72 h PFN1 treatment (100 µM) and lysates (60 ug/lane) subjected to Western blotting as previously described^31^. Blots were probed for ALOX5 (1:100, Abcam #ab169755) and GAPDH (1:2000, Cell Signaling, #3683).

#### Statistical Analysis

All statistical analyses were performed on raw data using GraphPad Prism 6 (GraphPad, SanDiego, CA, US). Where primary samples were used, the data was paired for statistical analyses to account for differences at baseline between samples. Data was tested for normality where possible (n > 6) and paired t-tests, Student’s t-tests, Mann Whitney t-tests, one-way ANOVA, repeated measures ANOVA or Friedman’s test were used as appropriate: the test used is indicated in the figure legends. All data is presented as mean ± SEM and as % change from control only for graphical presentation to account large variability between patient tissues at baseline. A p < 0.05 was considered statistically significant.

## Electronic supplementary material

Supplementary Info
