# Supplementary material for: Invasive trophoblast promote stromal fibroblast decidualization via Profilin 1 and ALOX5
Source: Sci Rep. 2017 Aug 18;7:8690. doi: 10.1038/s41598-017-05947-0 (PMC5562808; doi:10.1038/s41598-017-05947-0)

Invasive trophoblast promote stromal fibroblast decidualization via Profilin 1 and ALOX5

EM. Menkhorst[1,3^], ML. Van Sinderen[1^], K. Rainczuk[1], C. Cuman[1,3], A. Winship[1,2,3] and E. Dimitriadis[1,2,3*]

Supplementary Table 1. PFN1 regulation of decidualized HESC gene expression. HESCs were decidualized with E + MPA for 12 days then treated with 100µM PFN1 (or control) for 6h (n=4 pooled for array).

Gene symbol	Gene name	Fold regulation	p-value
ACE	Angiotensin I converting enzyme (peptidyl-dipeptidase A) 1	1.0701	0.59748
ACE2	Angiotensin I converting enzyme (peptidyl-dipeptidase A) 2	-1.6227	0.631998
ACTA2	Actin, alpha 2, smooth muscle, aorta	-1.6136	0.055612
ADM	Adrenomedullin	-1.3308	0.326346
ADRA1B	Adrenergic, alpha-1B-, receptor	-1.6836	0.134195
ADRA1D	Adrenergic, alpha-1D-, receptor	-1.0605	0.596688
ADRB1	Adrenergic, beta-1-, receptor	-3.7308	0.242764
AGT	Angiotensinogen (serpin peptidase inhibitor, clade A, member 8)	1.5939	0.87417
AGTR1	Angiotensin II receptor, type 1	-1.0946	0.603496
AGTR2	Angiotensin II receptor, type 2	-1.2133	0.300649
ALOX5	Arachidonate 5-lipoxygenase	-4.2358	0.083988
ARG2	Arginase, type II	-1.2202	0.526704
ATP2C1	ATPase, Ca++ transporting, type 2C, member 1	-1.0873	0.612559
ATP6AP2	ATPase, H+ transporting, lysosomal accessory protein 2	-1.2442	0.348456
AVP	Arginine vasopressin	1.6302	0.315794
AVPR1A	Arginine vasopressin receptor 1A	-1.2956	0.299781
AVPR1B	Arginine vasopressin receptor 1B	-1.2502	0.501759
BDKRB1	Bradykinin receptor B1	-1.1369	0.56641
BDKRB2	Bradykinin receptor B2	-1.2327	0.376215
BMPR2	Bone morphogenetic protein receptor, type II (serine/threonine kinase)	-1.2758	0.186456
CACNA1C	Calcium channel, voltage-dependent, L type, alpha 1C subunit	-1.0678	0.961448
CALCA	Calcitonin-related polypeptide alpha	-1.2133	0.300649
CAV1	Caveolin 1, caveolae protein, 22kDa	-1.2011	0.420584
CHRNA1	Cholinergic receptor, nicotinic, alpha 1 (muscle)	-1.2133	0.300649
CHRNB1	Cholinergic receptor, nicotinic, beta 1 (muscle)	-1.7253	0.268773
CLIC1	Chloride intracellular channel 1	-1.2663	0.399794
CLIC4	Chloride intracellular channel 4	-1.3383	0.249822
CLIC5	Chloride intracellular channel 5	-1.5007	0.355834
CNGA1	Cyclic nucleotide gated channel alpha 1	-1.3692	0.349697
CNGA2	Cyclic nucleotide gated channel alpha 2	-2.2372	0.257212
CNGA3	Cyclic nucleotide gated channel alpha 3	1.5808	0.423748
CNGA4	Cyclic nucleotide gated channel alpha 4	1.5594	0.74393
CNGB1	Cyclic nucleotide gated channel beta 1	-1.2133	0.300649
CNGB3	Cyclic nucleotide gated channel beta 3	-1.3395	0.463964
CPS1	Carbamoyl-phosphate synthase 1, mitochondrial	-1.3859	0.666555
DRD3	Dopamine receptor D3	-1.1212	0.45101
DRD5	Dopamine receptor D5	-3.519	0.346766
ECE1	Endothelin converting enzyme 1	-1.1442	0.605942

EDN1	Endothelin 1	1.3601	0.320205
EDN2	Endothelin 2	1.4951	0.426004
EDNRA	Endothelin receptor type A	-1.3932	0.391303
EDNRB	Endothelin receptor type B	-1.2895	0.694136
EPHX2	Epoxide hydrolase 2, cytoplasmic	-1.3983	0.249486
GCH1	GTP cyclohydrolase 1	1.1072	0.60154
GCHFR	GTP cyclohydrolase I feedback regulator	-1.5012	0.261939
GUCY1A3	Guanylate cyclase 1, soluble, alpha 3	-1.0945	0.692559
GUCY1B3	Guanylate cyclase 1, soluble, beta 3	1.1075	0.540619
HIF1A	Hypoxia inducible factor 1, alpha subunit (basic helix-loop-helix transcription factor)	-1.1128	0.569469
ITPR1	Inositol 1,4,5-trisphosphate receptor, type 1	-1.4677	0.407485
ITPR2	Inositol 1,4,5-trisphosphate receptor, type 2	-1.2765	0.348682
KCNJ8	Potassium inwardly-rectifying channel, subfamily J, member 8	-1.1934	0.706113
KCNMA1	Potassium large conductance calcium-activated channel, subfamily M, alpha member 1	-1.2887	0.430315
KNG1	Kininogen 1	-1.7483	0.663066
MYLK	Myosin light chain kinase	-1.329	0.152031
MYLK2	Myosin light chain kinase 2	2.5757	0.242757
MYLK3	Myosin light chain kinase 3	-1.3714	0.615145
NOS3	Nitric oxide synthase 3 (endothelial cell)	-1.6387	0.053471
NOSIP	Nitric oxide synthase interacting protein	-1.3045	0.32325
NOSTRIN	Nitric oxide synthase trafficker	-2.6417	0.56862
NPPB	Natriuretic peptide B	-1.5897	0.369638
NPPC	Natriuretic peptide C	-2.286	0.215343
NPR1	Natriuretic peptide receptor A/guanylate cyclase A (atrionatriuretic peptide receptor A)	-1.1752	0.740764
NPY1R	Neuropeptide Y receptor Y1	-1.0728	0.945792
P2RX4	Purinergic receptor P2X, ligand-gated ion channel, 4	-1.2243	0.419851
PDE3A	Phosphodiesterase 3A, cGMP-inhibited	1.2616	0.699758
PDE3B	Phosphodiesterase 3B, cGMP-inhibited	-1.0184	0.941464
PDE5A	Phosphodiesterase 5A, cGMP-specific	-1.1613	0.47685
PLCG1	Phospholipase C, gamma 1	-1.4033	0.31926
PLCG2	Phospholipase C, gamma 2 (phosphatidylinositol-specific)	-1.2108	0.672096
PRKG1	Protein kinase, cGMP-dependent, type I	-1.1223	0.666086
PRKG2	Protein kinase, cGMP-dependent, type II	-1.0374	0.732681
PTGIR	Prostaglandin I2 (prostacyclin) receptor (IP)	-1.0853	0.758162
PTGS1	Prostaglandin-endoperoxide synthase 1 (prostaglandin G/H synthase and cyclooxygenase)	-1.0717	0.660391
PTGS2	Prostaglandin-endoperoxide synthase 2 (prostaglandin G/H synthase and cyclooxygenase)	-1.4844	0.805191
REN	Renin	1.2777	0.942084
S1PR1	Sphingosine-1-phosphate receptor 1	-1.137	0.537154
SCNN1A	Sodium channel, nonvoltage-gated 1 alpha	-1.0155	0.627487
SCNN1B	Sodium channel, nonvoltage-gated 1, beta	-1.2133	0.300649
SCNN1G	Sodium channel, nonvoltage-gated 1, gamma	-1.0675	0.647292
SLC7A1	Solute carrier family 7 (cationic amino acid transporter, y+ system), member 1	-1.3564	0.30391
SPHK1	Sphingosine kinase 1	-1.1875	0.869492
SPHK2	Sphingosine kinase 2	-1.0165	0.748992
UTS2	Urotensin 2	-1.8281	0.655895
UTS2R	Urotensin 2 receptor	-3.0634	0.12733
ACTB	Actin, beta	-1.01	0.89812
B2M	Beta-2-microglobulin	1.1095	0.629779
GAPDH	Glyceraldehyde-3-phosphate dehydrogenase	-1.0892	0.689445

HPRT1	Hypoxanthine phosphoribosyltransferase 1	-1.021	0.908836
RPLP0	Ribosomal protein, large, P0	-1.0245	0.845745

Supplementary Figure.

Actual PRL concentration from Figure 1A&B.

a. HESCs (n=4) were treated with E for 14 days and CM treatments (HEK293, HTR8[/SVneo], EVT) included from day 7. PRL secretion was measured on day 14. EVT CM significantly increased PRL secretion by HESCs compared to control, HEK293 or HTR8/SVneo CM **b.** HESCs (n=4) were treated with E+MPA for 14 days with CM treatments (Hek293, HTR8, EVT) included from day 7. PRL secretion was measured on day 14. EVT CM significantly increased PRL secretion by HESCs compared to HEK293 CM. *p<0.05. a,b: Friedman test.

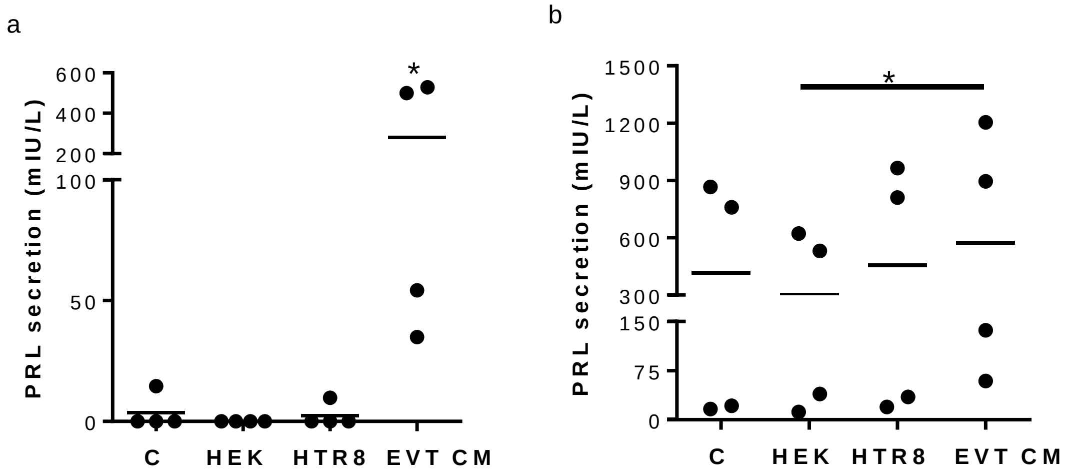

Supplement: Supplementary file 1 — Supplementary Info [file 41598_2017_5947_MOESM1_ESM.pdf]
